# A Service Evaluation on How Mental Health of Learning Disability (MHLD) Services Across Kent Respond to Dementia Referrals

**DOI:** 10.1192/bjo.2026.11577

**Published:** 2026-06-30

**Authors:** Yuanchao Xue

**Affiliations:** Kent and Medway Mental Health NHS Trust, Canterbury, United Kingdom. Oxford Health NHS Foundation Trust, Oxford, United Kingdom

## Abstract

**Aims:**

People with intellectual disability have a higher risk of developing dementia, yet assessment and diagnostic pathways can vary across services and regions. This service evaluation aims to evaluate how dementia referrals for people with intellectual disability are processed within Mental Health of Learning Disability (MHLD) services across Kent and identify opportunities to standardise referral and care pathways.

**Methods:**

A retrospective review was conducted of all referrals received by MHLD services in Kent from June 2022 to May 2023. Referrals were included if they contained themes suggestive of cognitive decline: changes in memory or function, forgetfulness, and confusion. Data were collected on referral source, demographics, reason for referral, acceptance or refusal, assessing discipline, follow-up plans, diagnoses at initial assessment and at 3 months. Referrals were analysed by region.

**Results:**

A total of 47 referrals met inclusion criteria. Acceptance rates varied by region and accepted referrals were younger on average than refused referrals across all regions. Down’s syndrome was present in a substantial proportion of accepted cases. Regional variation was observed in diagnostic pathways. Initiation of medication for dementia varied by clinician with consultants more likely to commence treatment than trainees.

**Conclusion:**

This evaluation highlighted regional variation in diagnostic pathways for people with intellectual disability and suspected dementia. The variation suggests a need for a standardised dementia referral and diagnostic pathway, guidance on in-house diagnosis, enhanced training for clinicians and allocation of wider resources to improve equity, efficiency and quality of care.

